# Post-training and mentorship experiences of KidzAlive-trained healthcare workers at primary healthcare facilities in KwaZulu-Natal, South Africa

**DOI:** 10.4102/phcfm.v12i1.2109

**Published:** 2020-06-29

**Authors:** Chipo Mutambo, Kemist Shumba, Khumbulani W. Hlongwana

**Affiliations:** 1 Discipline of Public Health Medicine, School of Nursing and Public Health, University of KwaZulu-Natal, Durban, South Africa; 2 Discipline of Psychology, School of Applied Human Sciences, University of KwaZulu-Natal, Durban, South Africa

**Keywords:** human immunodeficiency virus, capacity building, healthcare workers, child-centred care, quality of care

## Abstract

**Background:**

KidzAlive, a multicomponent, child-centred capacity building model was adopted by South Africa’s National Department of Health to address the challenges of quality of care among HIV+ children. This model involves training and mentoring healthcare workers (HCWs) on a child-centred care approach of communicating with children and their primary caregivers (PCGs). This study explored HCWs’ post-training experiences after a 6-months implementation period.

**Aim:**

To evaluate the KidzAlive model as a healthcare approach that seeks to improve the quality of HIV care among children.

**Setting:**

The study was conducted in 20 PHC rural and urban facilities across four districts in KwaZulu-Natal.

**Methods:**

Interviews were conducted by trained interviewers who followed a structured interview guide. These were audio-recorded, transcribed, and imported into NVivo 10 software for thematic analysis. Thematic analysis was used to develop a coding framework from the participant’s responses.

**Results:**

Five themes, namely: (1) increased healthcare worker knowledge, skills and confidence to provide child-friendly HIV services to children; (2) increased involvement of HIV + children in own healthcare journey; (3) the involvement of primary caregivers in children’s healthcare journey; (4) improved health outcomes for HIV + children; and e) transformation of the PHC environment towards being more child-friendly.

**Conclusion:**

The findings present preliminary evidence of successful KidzAlive trained HCWs’ buy-in of KidzAlive intervention. KidzAlive has been well integrated into current service delivery processes in PHC facilities. However, more rigorous research is warranted to fully understand the impact of this intervention on children and their primary caregivers.

## Introduction

South Africa bolsters the world’s largest human immunodeficiency virus (HIV) epidemic, carrying 19% of the global share of people living with HIV, 15% of new infections and 11% of acquired immunodeficiency syndrome (AIDS)-related deaths.^1,2,3^ Of the total number of people living with HIV in South Africa, an estimated 360 000 (0.5%) are children aged 0–14 years, with about 12 000 being new infections emanating from mother-to-child transmission.^3^ However, there has been a marked annual decline in the number of new HIV infections among children aged 0–14 years from 490 000 in the year 2000 to 25 000 in 2010 to 13 000 in 2017.^1,4^ Between 2010 and 2018, approximately 1 400 000 HIV infections among children were averted because of the implementation of prevention of mother-to-child transmission (PMTCT) programmes.^3^ These programmes have reduced the risk of infant HIV infection from an estimated 8% in 2008 to 3.5% in 2010, 2.6% in 2012–2013^5^ and 1.4% in 2015.^3,6^

Despite all the PMTCT successes, new HIV infections continue to occur, a phenomenon that has been attributed to low early infant diagnosis (EID) coverage.^7,8^ Studies report severe reductions of follow-up testing rates of HIV-exposed babies tested at birth from 89% to less than 45% at 10 weeks.^9,10^ One recent study conducted at the Mangaung University Community Partnership Programme Community Health Centre (MUCPP CHC) in the Free State province, South Africa, reported that only HIV-exposed infants (87.6%) received an HIV-1 polymerase chain reaction (PCR) test at birth, of whom 87.2% tested HIV-negative.^11^ Of the infants who tested HIV-negative at birth, 44.3% had a repeat PCR at 10 weeks, of whom 1.4% tested HIV-positive.^11^ The reduction in follow-up PCR testing of HIV-exposed infants translates to missed opportunities for case finding of babies who seroconvert at a later stage and those who are infected through breast feeding. As a result, these missed babies are never diagnosed. Unfortunately, they present later with serious and fatal AIDS-related illnesses.^12,13^ This challenge has resulted in a new epidemic of ‘slow progressors’ who have been loosely defined as perinatally infected older children or adolescents who have been able to survive without HIV medication.^12^ Research suggests that these slow progressors account for one-third of older children and adolescents living with HIV in high-burden settings.^12,14^

In addition to the challenges related to HIV case finding, children diagnosed and linked to care face unique challenges, including low disclosure rates, non-adherence to antiretroviral therapy (ART) and loss to follow-up.^13^ Disclosure, though often overlooked by HIV programmes, is one of the most important barriers to children’s adherence and retention in care.^15^ Research suggests that children who are aware of their HIV-positive status are more likely to adhere to medication and take action to increase control over their health.^16,17^ However, primary caregivers (PCGs) of children living with HIV are often unwilling to disclose to children under their care, because of, among other things, fear to trigger emotional distress in children.^13,15^ Most adults believe that children cannot handle the ‘bad’ news of an HIV-seropositive status.^15,18,19,20,21^ In South Africa, disclosure to children is deterred by existing paternalism, whereby the PCGs of HIV-positive children usually control the communication between healthcare workers (HCWs) and children under their care, which limits the HIV content shared with children.^15^ As a result, children living with HIV sometimes have limited power over what information, how, when and where they are disclosed to regarding their HIV status.^22,23^ Studies have shown that PCGs’ interference in HIV information flow between the HCW and a child is indicative of their lack of knowledge of HIV and child-friendly language to answer children’s difficult questions.^13,22,24,25^ Similarly, some studies suggest that front-line HCWs also lack the expertise to confidently practise child-centred HIV counseling, help PCGs disclose HIV-seropositive status to children or provide ongoing psychosocial support that addresses treatment adherence and sexual reproductive health.^15,20,26,27,28^

To address the challenges discussed above, child-centred HIV care approaches have been proposed.^13,29,30^ These approaches stem from patient-centred care, which the Institute of Medicine (IOM) defines as ‘Providing care that is respectful of, and responsive to, individual patient preferences, needs and values, and ensuring that patient values guide all clinical decision.’^29,31,32,33^ In the context of HIV, child-centred care approaches address the barriers to achieve UNAIDS’ 90–90–90 target by introducing innovative approaches that encourage child participation, age-appropriate information sharing, feedback from children and the modification of the healthcare environment to create a child-friendly ambience that dispels fear and stigmatisation of children living with HIV,^13,24,29,33,34^ which the KidzAlive model (KidzAlive) seeks to achieve. KidzAlive is a multi-component child-centred capacity building package for front-line HCWs delivering HIV care, psychosocial and wellness support to children aged 0–12 years living with HIV and AIDS.^29,35^ KidzAlive is driven by front-line HCWs providing HIV care to children, community-based organisations (CBOs) and primary healthcare (PHC) facilities in South Africa, using age-appropriate tools, resources and guidelines.^29^ The goal is to ensure that every child is provided with HIV care by a KidzAlive-trained HCW and the child is fully and continously engaged throughout his or her own healthcare to deal with emotional adjustments required for living with HIV. The KidzAlive intervention has been endorsed by the National Department of Health (NDoH) and since been included in the current adherence guidelines,^17^ disclosure guidelines^36^ and other handbooks that are used by HCWs providing HIV services to children in PHC settings.

From the year 2006 to 2019, 1411 HCWs from 422 PHCs in 17 districts from all the nine provinces of South Africa have participated in the KidzAlive programme and a full roll-out in all PHC facilities in South Africa is envisaged by 2020. Despite these plans, KidzAlive has not been formally evaluated and the decision to scale up has mainly been informed by the anecdotes of informal positive feedback from KidzAlive-trained HCWs. Therefore, subjecting this intervention into empirical research was considered a worthwhile exercise to justify the roll-out and/or improvements into the model.

## Overview of the KidzAlive intervention

KidzAlive is one of Zoë-life’s (South African-based non-governmental organisation [NGO] based in Durban, KwaZulu-Natal [KZN]) health promotion-oriented interventions. Its core business is to develop innovative solutions for addressing health and social challenges affecting children and adolescents. Zoë-life was founded in 2004 as a technical support partner to the NDoH in the area of HIV management among children and adolescents. Developed in 2006, KidzAlive is a differentiated care model that was conceived as a panacea to the problem of children being left behind in the country’s HIV response (see www.kidzalive.co.za). Its child-centred content is informed by child development theories, including Bandura’s social learning theory,^37^ Piaget’s cognitive developmental theory^38^ and Vygotsky’s sociocultural theory.^39,40^ These psychological theories are augmented by the Play Therapy Theory.

Over the years, Zoë-life’s technical support has included mainstreaming KidzAlive’s child-centred care approach into PHC and CBO settings, developing resources and training and mentoring front-line HCWs delivering HIV services to children. In 2015, KidzAlive was incorporated in the current adherence guidelines for HIV, TB and NCDs^17^ and the National Disclosure Guidelines for Children and Adolescents,^36^ which has contributed to its endorsement as the main differentiated care model for children by the South African NDoH.

## The components of the KidzAlive intervention

KidzAlive has three main components: KidzAlive HCW capacity building (training and mentorship), KidzAlive talk tool storybook and child-friendly spaces.

### KidzAlive capacity building

#### KidzAlive healthcare worker training

To select HCWs to participate in a deductive 5-day classroom training programme by two Zoë-life technical advisors, Zoë-life is guided by district management teams and PHC managers who nominate two HCWs per facility (nurses and HIV counsellors) already providing HIV testing and counselling, disclosure counselling, ART initiation and adherence counselling to children. These KidzAlive technical advisors are nurses and social workers with advanced qualifications and experience in HIV care and support for children. The training generally covers child rights, play therapy techniques, techniques for communicating with children, support mechanisms for PCGs, child-friendly spaces, stages of childhood development and dealing with children in distress.

#### KidzAlive mentorship

The KidzAlive technical advisors (now referred to as the mentors) commence with mentorship soon after the training. The group of 26 HCWs that they would have trained are split into two equal groups, each HCW is required to book eight child–PCG pairs to be provided with child-centred HIV care during the process of mentorship. Of these eight child–PCG pairs, three are provided with care by the mentor during a process called ‘preceptorship’ where the mentor demonstrates the care process to the HCW. The remaining child–PCG pairs receive care from the KidzAlive-trained HCW under the supervision of a mentor.

The competency of the KidzAlive-trained HCW is assessed by the mentor, using a KidzAlive mentorship checklist to rate the mentee’s aptitude in specific child-friendly skills. The KidzAlive-trained HCW is deemed competent if the mentor is satisfied that they have adequately demonstrated mastery of the key competencies taught during the 5-day training and preceptorship, with a minimum average score of 65%. Mentees are further given constructive feedback on areas requiring improvement. If the mentee scores less than 65%, she or he is deemed incompetent and the mentorship process is repeated until the standard is met.

#### KidzAlive talk tool storybook (talk tool)

KidzAlive-trained HCWs are provided with a colourful cartoon-based health education tool known as the talk tool for use while providing HIV services to children. The talk tool has the story side facing the child-friendly HCW (narrative text of the story), while the opposite side faces the child, showing a colourful cartoon illustration of the story being narrated by the HCW. When providing HIV care to the child in the child-friendly space, the KidzAlive-trained and mentored HCW sits opposite to the child and positions talk tool on a flat surface (usually a table).

The talk tool is characterised by visually telling and catchy illustrations, the story of *Sibusiso the Frog*. The story’s three protagonists are *Sibusiso the Frog*, his grandfather *Mkhulu Noah* and *Nurse Thelma*. In the story, *Sibusiso* learns about HIV, coping with stigma, and the importance of adherence to treatment. Both *Mkhulu Noah* and *Nurse Thelma* provide the necessary social support structure to *Sibusiso* in managing his HIV-seropositive status. This story uses age-appropriate language, for example, referring to medication as ‘Goodnight medicine that put germs to sleep’, HIV as a ‘Germ in your body’ and CD4 cells as ‘Soldiers that fight the germs’. During KidzAlive training, HCWs are taught about the child-friendly terminology to use with children of different ages and disclosure status. Recently, the talk tool was digitised into an *App*, which is being scaled up in KwaZulu-Natal and Gauteng provinces (https://www.youtube.com/watch?time_continue=6&v=qPy5XIxiXzk).

#### Child-friendly spaces

During KidzAlive training, HCWs are trained on how to create child-friendly environments or spaces in PHC facilities. For improved quality of care, children are encouraged to fully utilise these spaces during their routine visits to PHC facilities. Following the KidzAlive training, HCWs are provided with a child-friendly space toolbox to create a simple child-friendly space. These child-friendly spaces vary in size and include two colourful children’s chairs, a small table, toys and colour-in templates with characters from the KidzAlive talk tool. Children can decorate the space using their artwork crafted during previous consultations. In these spaces, KidzAlive-trained HCWs use their newly acquired play therapy skills to engage the children during healthcare consultations and promote positive healthcare experiences (https://www.youtube.com/watch?v=LN0pbLsMgJk).

## Research methodology

### The aim of the study

This study explored post-training and mentorship experiences of KidzAlive-trained HCWs providing HIV care to children at PHC facilities in KwaZulu-Natal, South Africa, with the overall aim of improving the quality of care.

### Research design

We adopted a qualitative explorative design rooted in the interpretive paradigm^41^ to explore the experiences of KidzAlive-trained and mentored HCWs.

### Study setting

The KwaZulu-Natal Provincial Department of Health continues to receive technical support from Zoë-life to mainstream the KidzAlive approach into HIV service delivery for children through the Unfinished Business (UB) initiative. Unfinished Business was initiated in 2015 to accelerate the province’s efforts towards achieving the 90–90–90 targets for children aged 0–19 years. Through UB, 400 HCWs from 180 PHC and CBOs in four districts in KZN (uMgungundlovu, eThekwini, uMkhanyakude and Zululand) have been trained, mentored and provided with KidzAlive job aids (talk tool). Through KidzAlive, to date, 80 PHCs have created child-friendly spaces. This study was conducted under UB, as an evaluation exercise to explore the post-training experiences of KidzAlive-trained HCWs in 40 PHCs that were receiving support from Zoë-life between 2018 and 2019.

### Study participants

The study was conducted among 40 HCWs from the 40 PHC facilities participating in KidzAlive programme. This study was conducted 6 months after the KidzAlive training and mentorship exercise, which ended on the last week of September 2018.

### Selection of participants

We purposively selected HCWs (*n* = 80) from PHC facilities that were earmarked to receive KidzAlive support from Zoë-life in September 2018 through the UB. The HCWs who were selected for KidzAlive training and mentorship were those whose main responsibility was to provide HIV services to children including HIV education, HIV counselling and testing (HCT), disclosure counselling and medication adherence support. The HCWs were nominated by their PHC managers.

We endeavoured to interview the 80 KidzAlive-trained HCWs or until data saturation. However, because of resource constraints, we conveniently reduced our sample size to 20 KidzAlive-trained HCWs from 20 PHCs. These KidzAlive HCWs included 13 HIV counsellors and seven nurses from 10 urban-based PHC facilities (eThekwini and uMgungundlovu) and 10 rural-based PHC facilities (Zululand and uMkhanyakude), respectively. Of each category (rural and urban), we further purposively selected five low-volume and five high-volume facilities.

### Data generation

Four research assistants (RAs) with prior training and experience in qualitative research were recruited and given refresher training on qualitative data collection by the first and second authors (C.M. and K.S.) prior to commencing the fieldwork. The RAs were also familiarised with the KidzAlive intervention to contextualise the questions and to allow them to perform deep probes. They were allocated into two teams led by C.M. and K.S. Each team collected data from two districts (10 facilities).

Following their training, RAs were tasked with scheduling 1-h long interviews with the participants. The KidzAlive-trained HCWs gave written and verbal consents for interviews and audio records, respectively. All participants were assured of privacy and confidentiality of their personal information, including their health facilities. The RAs conducted one-on-one structured interviews with five KidzAlive-trained HCWs from each district. During the interviews, the RAs took field notes and recorded each interview using a digital audio recorder. The interviews were conducted in English because the HCWs were able to communicate fluently in English. In addition to the interviews, C.M. (lead researcher) also used a diary to record some of her observations during HCW mentorship and on-site visits to all the 20 facilities.

### Data analysis

Thematic data analysis was iteratively conducted manually by C.M. and K.S. Data were transcribed into Excel and then uploaded on NVivo version 10 for coding. Thematic analysis was used inductively to code the data, and this proceeded in three phases. The first phase involved the analysis of data, which resulted in the development of a preliminary code book. This code book was based on topics that were frequently cited. Key codes were then generated and amendments to the initial code book were made iteratively as both researchers analysed the rest of the data, resulting in the adoption of the finalised code book.

The second phase involved the construction of overarching categories, whereby similar codes were grouped together to identify emerging patterns, relationships, contrasts and paradoxes between the codes. The last phase involved the organisation of the categories into themes and sub-themes to address the main research question. Verbatim quotes were used to substantiate the findings. Participants’ and facilities’ names are hidden for privacy and confidentiality purpose; instead, two letter abbreviations of the district were used as codes.

### Trustworthiness

Guba (1981) identifies four important criteria to enhance trustworthiness in any qualitative inquiry. These are credibility (internal validity), transferability (external validity generalisability), dependability (reliability) and confirmability (objectivity).^42^ To ensure credibility, we paid attention to negative cases whereby the KidzAlive-trained HCWs cited negative experiences and/or viewpoints from others in relation to the KidzAlive intervention; we retrieved and presented verbatim quotes from audio recordings and analysis of the data by different members of the research team to fulfill the analyst triangulation criteria.^43,44^ Dependability was achieved through independent co-coding of the data by two researchers (C.M. and K.S.), ensuring that the analysis was grounded in data with the supportive verbatim quotes in all the themes and sub-themes presented in the findings. Transferability of the findings was ensured through the clear exposition of study participants and providing a thick description of the background, methods and findings. Confirmability was ensured by documenting the entire research process using audio recordings, field notes and active participation of the research team in the data analysis. Furthermore, the research data have been kept in a file that can be retrieved.

### Ethical consideration

Ethical clearance for this study was obtained from the University of KwaZulu-Natal’s Biomedical Research Ethics Committee (BREC) (Approval No. BE 298/18) and the Directorate for Health Research and Knowledge Management (KZ_201809_011). Gatekeeper permissions were obtained from the HIV, AIDS and STIs Directorate at the KwaZulu-Natal Department of Health Provincial Office and the Zoë-life Directorate, respectively. Written informed consent was sought from all the adult participants prior to conducting the respective interviews. Parental consent was also sought in writing. Lastly, assent was obtained from all the children who participated in the study using a child-friendly story-based information sheet and assent form.

## Findings

### Demographic data

A total of 20 KidzAlive-trained HCWs from 20 PHC facilities were interviewed, and 90% of whom were women and participants’ ages ranged between 25 and 45 years. Two-thirds of the participants were HIV counsellors, with the remaining one-third being nurses. The KidzAlive-trained HCWs had worked with children for an average of 4 years (range 3–5 years).

Data analysis revealed five themes. These were (1) increased HVW knowledge, skills and confidence to provide child-friendly HIV services to children; (2) increased involvement of HIV-positive children in own healthcare journey; (3) the involvement of PCGs in children’s healthcare journey; (4) improved health outcomes for children living with HIV and (5) transformation of the PHC environment towards being child-friendly.

#### Increased healthcare worker knowledge, skills and confidence to provide child-friendly human immunodeficiency services to children

The findings suggest that the KidzAlive HCWs were unaware of the need to engage and involve children, prior to the intervention. The goal of KidzAlive training and mentorship is to increase participants’ knowledge, skills and confidence to deliver child-centred HIV services.^29^ This knowledge includes communicating with children, engaging children and ensuring that they participate during the care consultation. Following this intervention, the KidzAlive-trained HCWs reported to have gained knowledge of appropriate terminology to use when communicating with children of various ages during HIV care (P3, P5, P9, P11, P12–P20):

‘[*I*] didn’t know that I was supposed to involve children and even how to do it. This training enabled me to understand the importance of involving children when providing HIV services. This training included information on how to effectively communicate with children of different ages and to use the right words for the child to understand difficult concepts. Furthermore, the people from Zoë-life came to help us at the facility and taught us how to use the tools that they had given us during training.’ (31-year-old HIV Counsellor, UMG5 Clinic, uMgungundlovu District)

To enhance communication with children, the use of child-friendly job aids is encouraged.^29^ It was clear from the findings that the inclusion of job aids such as the talk tool marginally improved the communication process. Use of the talk tool enhanced the HCWs’ ability to provide age-friendly information and address children’s questions in a manner that increases the child’s understanding of HIV while keeping them engaged in the entire care process (P1–P7, P12, P14):

‘[*A*]t first, we were afraid to talk to children, we didn’t have the skills to communicate with children of different ages as we didn’t know which words to use to enhance their understanding. Now I can answer questions such as: “What is HIV?”, “What are ARVs?”, “How do ARVs work?”. Before KidzAlive, children would ask questions and I would use language that was too advanced for them to comprehend, such that it created more questions and confusion for the child. Now with KidzAlive, the story telling technique and the terminology used in the storybook, it is much easier to convey messages to children.’ (35-year-old, HIV Counsellor, ETH2 Clinic, eThekwini District)

Generally, the KidzAlive-trained HCWs reported that the KidzAlive job aids were adaptable, as they made it possible for them to adjust the conversations for different age groups, including the older children and PCGs (P1, P8, P9, P10, P20). The versatility of the talk tool was an important finding because the job aids are primarily developed to enhance the communication process, particularly with children:

‘[*I*]t’s not just the small children, even adolescents and PCGs especially gogos [*grandmothers*] of the children also enjoy the story and it makes them understand HIV and how it affects the body. It makes them understand the importance of taking medication.’ (45-year-old, Professional Nurse, UMG2 Clinic, uMgungundlovu District)

KidzAlive-trained HCWs were consistent in expressing their satisfaction with the content of KidzAlive trainings, highlighting that it had opened their eyes to the skills gaps militating against the provision of HIV services to children (P1–P20):

‘[*P*]reviously, I mean before this KidzAlive training, we were just talking to the PCGs of the child thinking that the child would not understand. Now I know better. The training provided by Zoë-life enabled us to understand what we were doing wrong and that we were depriving children of their right to health, and right to information. We now understand the importance of engaging with the child and explaining to them in a way that they understand. However, it has not been easy as I’m still learning and gaining confidence to talk to these children.’ (35-year-old Professional Nurse, ZUL2 Clinic, Zululand District)

Despite the positive feedback that HCWs gave to the training, inadequacy of the coaching from KidzAlive mentors remained a concern. Most participants specified that the mentorship process was inadequate and that they would prefer that mentors demonstrate cases to give the mentees an opportunity not only to learn, but also to gain more confidence (P2, P11, P17):

‘[*W*]e still need more mentorship because sometimes the mentors expect us to implement without demonstrating on practical cases and they just come to assess us. We need more coaching and demonstrations.’ (25-year-old HIV Counsellor, UMG2 Clinic, uMgungundlovu District])

KidzAlive-trained HCWs requested for HCW-focused psychosocial support during mentorship visits, which will give them an opportunity to debrief professional social workers on their experiences during training. Doing so can help them to cope with stress and build their resilience (P1–P12, P14, P16–P20):

‘[*W*]e meet children in very difficult circumstances, and this affects us. We need KidzAlive to include training on how to overcome this stress and these feelings because it affects us even when we go home. We also need to engage with our mentors to provide us with counselling on how best to deal with the difficult situations we face with children and their caregivers.’ (31-year-old HIV Counsellor, ETH1 Clinic, eThekwini District)

#### Increased involvement of human immunodeficiency virus-positive children in their healthcare journey

There was a convergence of opinions in that the KidzAlive talk tool had improved the way HCWs related with the children, resulting in children leaving healthcare facilities with no trauma or fear – rather with excitement about being able to look after their bodies in the same way as their new friend ‘Sibusiso’ (P1–P5, P7, P17–P20):

‘[*T*]he KidzAlive talk tool is the best tool for children I have seen since I qualified as a nurse. You can show the child the pictures and effectively discuss with the child about HIV, making it easy for them to comprehend. More so, the children and their PCGs enjoy the story and when they leave the health facility, they are excited to start looking after their bodies like Sibusiso the frog in the story.’ (45-year-old, Professional Nurse, UMG2 Clinic, uMgungundlovu District)

The age-sensitive information contained in the KidzAlive talk tool was echoed as one of the important strengths of the approach. Participants indicated that this tool increased child involvement in the provision of care (P1–P9, P15, P20). In addition, observations made during counselling sessions conducted in child-friendly facilities showed that storytelling and toys kept the child engaged in the whole process of providing care and reduced the fear and anxiety of being with an HCW:

‘[*J*]ob aids and child-friendly spaces are important for providing age-appropriate information to children. They help the child to participate and stay involved in the counselling process. Using play techniques and toys in the space helps to make-up stories and initiate a conversation with the child. The child-friendly spaces ensure that children participate in their healthcare, which was previously not the case because we would only talk to the child’s parent as we thought that the child was not mature enough to understand.’ (34-year-old, HIV Counsellor, ZUL5 Clinic, Zululand District)

There was consensus in that the KidzAlive intervention had increased children’s knowledge and understanding of HIV, the importance of taking medication and the consequences of non-adherence as well as supporting disclosure (P1–P20):

‘[*K*]idzAlive helps especially in the context of disclosure …highlighting the consequences of not adhering to treatment … it improves children’s understanding and promotes medication adherence if children have been provided with adequate health education.’ (25-year-old, HIV Counsellor, UMGU5 Clinic, uMgungundlovu District)

‘[*T*]he training helped us to motivate the child to take medication and have hope for their future … I say, “What do you want to do with your life after school?” and the child responds, “Yes, I take my tablets because I want to be a doctor.”’ (25-year-old, HIV Counsellor, UMG3, Clinic uMgungundlovu District)

The HCWs asserted that KidzAlive training made them more ‘friendly’ to, and approachable by children. Participants also confessed that children who were not seeking HIV care were spontaneously gravitating at these child-friendly spaces without an accompanying adult (P7, P9–P15, P20):

‘[T]he children have started to be open and friendly to me. The child-friendly space makes the children be fearless. Children now just randomly come to the child-friendly space to play even when they are not seeking care from me.’ (25-year-old, HIV Counsellor, UMGU5 Clinic, uMgungundlovu District)

#### Increased involvement of primary caregivers in children’s healthcare journey

All the KidzAlive-trained HCWs concurred that the training and mentorship programme equipped them with the relevant skills to provide psychosocial support to PCGs. They attested that it allayed key fears and concerns related to having their children tested, disclosed to and encouraged to adhere to medication (P1–P20):

‘[*M*]any of them [*PCGs*] had not disclosed to their children and they were concerned about telling them about their medication, fearing that the children would default on treatment.’ (32-year-old Professional Nurse, ETH3 Clinic, eThekwini District)

‘[*T*]he KidzAlive talk tool includes the “primary caregiver preparation” section whereby PCGs of children living with HIV are provided with counselling and education on why it is important to disclose to their children and how disclosure can help them to adhere to medication and participate in their own healthcare journey. We also provide the PCG with guidance on how to disclose, “which” words to use, “when” to disclose to the child and “how” to continue communicating with the child post disclosure.’ (25-year-old, HIV Counsellor, UMGU5 Clinic, uMgungundlovu District)

KidzAlive-trained HCWs reported an increase in PCGs’ willingness to consent to test their children, and take their children for treatment, medication collection and initiating disclosure to their children, following programme implementation. The HCWs also reported a reduction in missed appointments, particularly among children involved in adherence support groups (P1–P20):

‘[*M*]any PCGs now consent to have their children tested, especially after speaking to them about the benefits of testing children for HIV. You can even see that they now understand why it is important. Furthermore, parents that we have counselled have joined support groups and are now bringing children for drug-pickup [antiretroviral medication] to prevent defaulting.’ (31-year-old HIV Counsellor, UMG5 Clinic, uMgungundlovu District)

However, staff shortages and high patient congestion at healthcare facilities remained a hindrance to the optimal contribution of the KidzAlive programme. These two challenges limited the amount of time spent on each individual child and his or her PCG:

‘[*A*]s nurses, we must see all people living with HIV, yet at times we have no time to provide a comprehensive service to children using the Kidzlive talk tool. However, we use the skills taught during training to engage with children and their primary caregivers. However, the sessions often become too long and other patients start to complain.’ (32-year-old HIV Counsellor, ZUL1 Clinic, Zululand District)

Staff shortages resulted in involuntary staff rotation and working at a different department within the healthcare facility for a prolonged period. These adjustments disrupted the implementation of the KidzAlive intervention (P1, P2, P3, P9, P12, P20):

‘[*S*]taff rotations disrupt the implementation of KidzAlive, but we have no choice. When I am moved, no one is left to provide the service or someone new is deployed, which confuses the clients because they will need to get used to this new HCW and trust them to care for them. It could be better to train all of us [*HCWs working in PHC settings*] so that anyone who goes to that station for children is able to provide a consistent child-friendly service.’ (32-year-old HIV Counsellor, ZUL3 Clinic, Zululand District)

Lack of healthcare centre management support was highlighted as an important barrier to the implementation of the KidzAlive programme. Participants suggested that KidzAlive training be extended to PHC managers as a strategy to canvass their buy-in for adequate staff deployment and increased allocation of resources needed to effectively implement KidzAlive intervention (P1, P2, P3, P9, P12, P20):

‘[*M*]y manager doesn’t understand the programme and when I am using the tools, more time is consumed, and she complains that the health facility is congested. I must explain that providing services to children takes longer as I must counsel both parties (PCG and child) and ensure that they understand what I would be talking about. I think its best that the managers also receive training so that they understand the whole process and what it takes.’ (32-year-old HIV Counsellor, ZUL5 Clinic, Zululand District)

#### Improved health outcomes for children living with human immunodeficiency virus

KidzAlive-trained HCWs reported that children who were identified after the KidzAlive training and mentorship were recruited into support groups. They were provided with structured adherence support sessions, and continuous HIV and wellness education to ensure that they remain in care and are adherent to treatment. Newly initiated children who were recruited into adherence support groups with high viral loads were reported to have a marginally reduced viral load after the 6-month period. Furthermore, both the number of children presenting for HIV testing and the number of children disclosed to increase (P1, P3, P9, P12, P19, P20):

‘[*W*]e have formed adherence support groups for children, but they are still in their youthful stages. However, we are already seeing the results as the children involved in our support groups now have reduced viral loads, showing that they have understood the information we give and that they are adhering to their medication. This makes us happy.’ (36-year-old HIV Counsellor, UMG1 Clinic, uMgungundlovu District)

The study participants also reported that the implementation of KidzAlive helped to improve healthcare service delivery because PCGs could now bring their HIV-positive children to the healthcare facility. This helped HCWs to assess the children and recommend the appropriate treatment regimen:

‘[*P*]CGs now bring their children for drug pick-ups. Prior to the intervention, only PCGs were coming for drug pick-ups and it was hard for us to convince them to bring children to the clinic so that we could assess them and give them the right dosage.’ (39-year-old HIV Counsellor, UMK3 Clinic, uMkhanyakude District)

#### Transformation of the primary healthcare environment towards being child-friendly

KidzAlive-trained HCWs reported being unaware of the importance and value of creating a child-friendly healthcare environment, prior to the intervention implementation (P3–P9, P20):

‘[*C*]hild-friendly spaces have been vital to the provision of quality clinical services to children and it made me able to work well with caregivers, particularly regarding full disclosure about the child’s HIV- positive status. The child-friendly spaces have changed the clinic from being an ordinary place to one where children belong, and can take control of their health.’ (31-year-old HIV Counsellor, UMK2, eThekwini District)

Knowledge and skills transfer to colleagues after training HCWs is one of the expectations of training. Similarly, The KidzAlive-trained HCWs also reported that they had shared the new knowledge, skills and job aids they acquired from the KidzAlive programme with their peers who received them with enthusiasm (P1–P2, P6, P9, P12, P20).

‘We have shared the job-aids with our colleagues, and they are very impressed. They have hinted that they would also like to be trained.’ (33-year-old HIV Counsellor, UMK2 Clinic, uMkhanyakude District)

In this study, KidzAlive-trained HCWs reported that they had only seen child-friendly spaces in private clinics and hospitals, and hence, the KidzAlive child-friendly spaces had transformed an ordinary PHC environment to look more like a children’s ward at a private hospital, a shift that was appreciated by the local communities (P1–P20):

‘[*I*] had only seen children’s play areas at private clinics and private hospitals. Now even those of us in the community have a private hospital experience at our local clinic because of KidzAlive. The community is commenting when they see the child-friendly space and tell us that they like the way we are trying to include their children.’ (42-year-old HIV Counsellor, ETH4 Clinic, eThekwini District)

Despite the known benefits of child-friendly spaces, the shortage of physical space for creating child-friendly spaces remains a challenge. Based on our observations during field visits, only 10 (25%) out of the 40 sites included in the study had a standard child-friendly space, and 14 (35%) created temporary spaces in shared counselling rooms, while 16 (40%) facilities did not have any space at all.

There were concerns regarding the child–PCG pair’s confidentiality being compromised in PHCs where child-friendly spaces are in a shared office (P1, P7, P9):

‘[*W*]e have experienced challenges with finding space to enact child-friendly spaces in this facility because there simply isn’t much space. Consequently, we have converted this counselling room which we share with other counsellors to establish a child-friendly corner. However, PCGs don’t like this as it is not private, and children can only access it when they are receiving HIV services from KidzAlive Trained HCWs.’ (33-year-old HIV Counsellor, UMG2 Clinic, uMgungundlovu District)

Lastly, KidzAlive-trained HCWs reported that the current KidzAlive curriculum was not inclusive and did not sufficiently cater for children living with disability (e.g. deaf, dumb and blind children). They suggested that the training curriculum and tools be augumented to such that they can be used for children with disabilities who continue to be an underserved population by HIV-focused healthcare programmes:

‘[*W*]e encounter a substantial number of children that are infected with HIV and have disabilities. Some can’t see, hear, or speak or both. Maybe next time we can get training on how to deal with such children.’ (36-year-old HIV Counsellor, ZUL2 Clinic, Zululand District)

## Discussion

From a public health perspective, the KidzAlive training and mentorship intervention support the NDoH’s goals to reach its UNAIDS 90–90–90 targets. The realisation of these targets in the context of children requires child-friendly impactful innovations, which are economically sustainable and embraced by all stakeholders. The findings of this study indicate that the KidzAlive intervention partly fulfils this criterion, given its reception by key stakeholders, as evidenced by its rapid integration into the existing healthcare service delivery processes in the target PHC facilities. In addition, KidzAlive seems to be acceptable and simple to implement and integrate into existing HIV services of low-resource settings.

Most KidzAlive-trained HCWs appreciated the mentorship and coaching process, as they saw it as an opportunity for them to make mistakes, be corrected, seek guidance and share ideas with mentors. Similarly, the value of on-the-job learning through mentorship was observed in the Zenith Trial in Zimbabwe, where HCWs were provided with on-the-job mentoring, which did not only result in HCWs’ efficient management of problems, but also contributed to long-term job satisfaction and increased commitment to healthcare service delivery.^45^ It was noted that correctly implemented mentorship was appreciated. Similar findings were reported in a study conducted in the Extending Quality Improvement for HIV/AIDS Project (EQUIP) project, Malawi, whereby mentors spent substantial time demonstrating procedure, while mentees were observing, thereby improving the mentees’ learning, skills development and confidence to perform difficult tasks.^46^

High patient burden and staff shortages are a pervasive problem in resource-limited PHC settings, and the PHCs in KZN are no exception. In addition, PHC management buy-in was flagged as a pervasive problem across all facilities, and managers were preoccupied with the amount of time required to implement and achieve the objectives of the KidzAlive programme. As a result, HCWs hardly provide the full KidzAlive care, thereby compromising the quality of care for children. Perhaps, the developers of KidzAlive should consider modifying the intervention for high patient burden scenarios through training other lower level cadres, such as community care workers, HIV counsellors, social auxiliary workers and nursing assistants. This will marginally reduce both patient waiting times and consultation times per patient attended by the overburdened nurse while maintaining the quality of care. In addition, training PHC managers on KidzAlive can contribute to increasing their buy-in, appropriation of the KidzAlive model and allocation of resources towards making it a reality. This may culminate into the reduction of staff rotations for KidzAlive-trained HCWs and task shifting of their additional responsibilities to other staff members.

Some KidzAlive-trained HCWs reported that they found it emotionally taxing to continuously deal with difficult cases and trauma involving vulnerable and very sick children. They requested for an expansion of the mentorship support package to include a psychosocial support component, specifically to deal with HCW trauma, so that they can debrief and receive counselling to help them cope with their work. There is limited literature on this subject in South Africa. Apparently the current healthcare system is not sufficiently responsive to the mental well-being of its health workforce, especially at the PHC level. This is concerning for a country with the highest HIV burden in the world, 90% of whom are receiving constant chronic care and support from PHCs that are plagued with staff shortages, high caseload and poor work morale.^47^ The absence of trauma and resilience support can result in high provider stress levels, thereby negatively impacting on the functioning of the healthcare system with respect to retention, performance, productivity, safety, demand and quality of care and job satisfaction.^48,49^ Although very little is being performed in low-resource PHC settings to promote HCW resilience, studies propose that trauma-sensitive healthcare centres be created, with the provision of psychosocial interventions to HCWs becoming a priority.^50^

The absence of child-friendly job aids to assist HCWs providing HIV management services is widely reported as one of the biggest barriers to quality HIV care for children.^20,28,51,52^ It is for this reason that the KidzAlive child-friendly job aid was positively received and is regarded as the mainstay of the KidzAlive intervention in South Africa. Storytelling and play therapy techniques taught during the KidzAlive training and reinforced during mentorship, and made it easier for the KidzAlive-trained HCWs to have age-appropriate conversations with children, explaining difficult concepts asked by children.

In addition, the KidzAlive talk tool also provided the KidzAlive-trained HCWs with a stepwise guide to follow when providing HIV services ranging from provider-initiated counselling and testing, initiation, adherence and viral load monitoring to disclosure. These findings are consistent with those reported in Namibia where a similar child-friendly job aid called the ‘Disclosure Storybook’ was well accepted for use to support status disclosure for children aged 7–12 years.^16,52^ Despite the high regard given to the KidzAlive job-aids and materials used during the consultation process, HCWs reported that the intervention required more time, which they did not have, because of high patient volume.

In addition, the unavailability of space in PHC facilities poses a huge problem, thereby hindering the successful and effective implementation of child-friendly spaces in PHC settings. The study highlighted that permanent spaces were difficult to create. This is an important finding that can inform the developers of the intervention to investigate what these temporary spaces look like and how they can be improved for the maximum benefit of children.

Although the KidzAlive-trained HCWs acknowledged that KidzAlive was comprehensive, they suggested that the developers should expand the content to suit deaf, dumb and blind children, as these continue to be left behind by health programmes.^53^ This was a profound suggestion as HIV health programmes continue to ignore the high prevalence of disability among children vertically infected with HIV. Consistent with the findings from other countries in sub-Saharan Africa, a study conducted in Malawi reported a disability prevalence of 33.3% among children living with HIV, with the odds of disability being eight times higher in the HIV-infected child compared to their uninfected counterparts.^54^ In spite of the increased vulnerability, these children remain an afterthought in many HIV programmes.^53,55^ Children living with both disabilities and HIV are at increased risk of developing disabling sequelae from long-term infection, co-morbidities and the side effects of their treatment.^53^

## Strengths of the study

To our knowledge, this study is the first to present the experiences of the KidzAlive-trained HCWs. Secondly, there were no notable differences in the challenges related to the implementation of KidzAlive in rural and urban facilities. This is indicative of the versatility of KidzAlive in PHC settings, which is an important finding that can support its scale-up to all PHC centres in South Africa.

However, there were some limitations to the study. Firstly, we did not interview end beneficiaries of KidzAlive, that is, children and their PCGs. This could have helped us to obtain multiple perspectives on the outcomes of the KidzAlive intervention and its acceptability by its end beneficiaries. Further research on the perspectives of the beneficiaries of KidzAlive is currently underway.

## Conclusion

The study provides data to help make adjustments that would improve the usefulness of KidzAlive in the existing PHC setting in South Africa. These improvements will contribute towards accelerating the NDoH’s thrust towards improving case finding of untested children, promote same-day initiation of ART, support children’s adherence to medication, increase disclosure rates and improve the health outcomes and quality of life of children living with HIV. This study contributes to the much-needed body of evidence to support the scale-up of this intervention while informing developers and future funders of the potential barriers to its success and effectiveness.
